# Facilities for macromolecular crystallography at the Helmholtz-Zentrum Berlin

**DOI:** 10.1107/S0909049512006395

**Published:** 2012-03-20

**Authors:** Uwe Mueller, Nora Darowski, Martin R. Fuchs, Ronald Förster, Michael Hellmig, Karthik S. Paithankar, Sandra Pühringer, Michael Steffien, Georg Zocher, Manfred S. Weiss

**Affiliations:** a Helmholtz-Zentrum Berlin für Materialien und Energie, Institute for Soft Matter and Functional Materials, Macromolecular Crystallography, Albert-Einstein-Strasse 15, D-12489 Berlin, Germany; b Swiss Light Source at Paul Scherrer Institut, CH-5232 Villigen PSI, Switzerland; c Freie Universität Berlin, Fachbereich Biologie, Chemie, Pharmazie, Institut für Chemie und Biochemie, AG Strukturbiochemie, Takustrasse 6, D-14195 Berlin, Germany; d Universität Tübingen, Interfakultäres Institut für Biochemie, Hoppe-Seyler-Strasse 4, D-72076 Tübingen, Germany

**Keywords:** macromolecular crystallography beamlines, high-throughput methods, crystal dehydration, UV radiation-damage-induced phasing, long-wavelength phasing

## Abstract

Three macromolecular crystallography (MX) beamlines at the Helmholtz-Zentrum Berlin (HZB) are available for the regional, national and international structural biology user community. The state-of-the-art synchrotron beamlines for MX BL14.1, BL14.2 and BL14.3 are located within the low-β section of the BESSY II electron storage ring. All beamlines are fed from a superconducting 7 T wavelength-shifter insertion device. BL14.1 and BL14.2 are energy tunable in the range 5–16 keV, while BL14.3 is a fixed-energy side station operated at 13.8 keV. All three beamlines are equipped with CCD detectors. BL14.1 and BL14.2 are in regular user operation providing about 200 beam days per year and about 600 user shifts to approximately 50 research groups across Europe. BL14.3 has initially been used as a test facility and was brought into regular user mode operation during the year 2010. BL14.1 has recently been upgraded with a microdiffractometer including a mini-κ goniometer and an automated sample changer. Additional user facilities include office space adjacent to the beamlines, a sample preparation laboratory, a biology laboratory (safety level 1) and high-end computing resources. In this article the instrumentation of the beamlines is described, and a summary of the experimental possibilities of the beamlines and the provided ancillary equipment for the user community is given.

## Abstract 2

The three macromolecular crystallography beamlines BL14.1, BL14.2 and BL14.3 at the BESSY II storage ring at the Helmholtz-Zentrum Berlin are described.

## 1. Introduction

Structural biology using X-ray crystallography requires the availability of powerful and versatile synchrotron-based X-ray diffraction beamlines and experimental stations to support the still growing demand for highly brilliant tunable-energy X-ray sources. Currently, the European structural biology community has access to nine synchrotron sources with a total of about 29 beamlines for macromolecular crystallography (MX) applications: BESSY II (Berlin, Germany, three beamlines), Diamond Light Source (Didcot, UK, five beamlines), ELETTRA (Trieste, Italy, one beamline), EMBL/DESY (Hamburg, Germany, three beamlines), ESRF (Grenoble, France, eight beamlines), KURCHATOV SNC (Moscow, Russia, one beamline), MAX II (Lund, Sweden, four beamlines), SLS (Villigen, Switzerland, three beamlines) and SOLEIL (Saint-Aubin, France, one beamline). Currently under construction or commissioning are three MX beamlines at the PETRA-III ring (Hamburg, Germany), one MX beamline at SOLEIL and one MX beamline at the ALBA synchrotron (Barcelona, Spain).

All of these beamlines are heavily used by the European and worldwide MX community. With these MX beamlines, the European synchrotrons contribute to about 33% of all macromolecular structures determined and deposited worldwide (http://biosync.sbkb.org/).

## 2. Facility description

### 2.1. BESSY II storage ring

The third-generation 1.7 GeV electron storage ring BESSY II of the Helmholtz-Zentrum Berlin für Materialien und Energie (HZB) is a dedicated light source for the production of ultrabright photon beams from the long-wavelength terahertz region to hard X-rays, located on the WISTA Science and Technology Campus in Berlin-Adlershof (Germany). Close to 50 beamlines with excellent energy resolutions offer a multi-faceted mixture of experimental opportunities at undulators, wigglers and dipole sources. Despite the fact that most beamlines at BESSY II utilize the XUV part of the electromagnetic spectrum (1–2000 eV), a number of hard X-ray beamlines have also been installed and operate successfully. The high-resolution EXAFS and photoelectron spectroscopy at high kinetic energies (HIKE) beamline KMC-1 (Schaefers *et al.*, 2007 ▶) and the microfocus X-ray absorption spectroscopy (microXAS) beamline KMC-2 (Erko *et al.*, 2001 ▶) are using classical bending magnets as sources, which have a critical energy of 2.5 keV. More flux and brilliance at higher X-ray energies can be gained from superconducting magnet structures operated at the liquid helium temperature of 4 K. The magnetic scattering beamline MagS uses a superconducting 13-pole, 7 T wiggler (Dudzik *et al.*, 2006 ▶), whereas the material science beamline BAMline (Gorner *et al.*, 2001 ▶) and the micro-focus beamline for microXAS and microXRD (Erko *et al.*, 2004 ▶) are using a shared 7 T wavelength shifter (7T-WLS) (Budker Institute of Nuclear Physics Novosibirsk, Russia).

### 2.2. The X-ray source for MX

**Figure 1:**
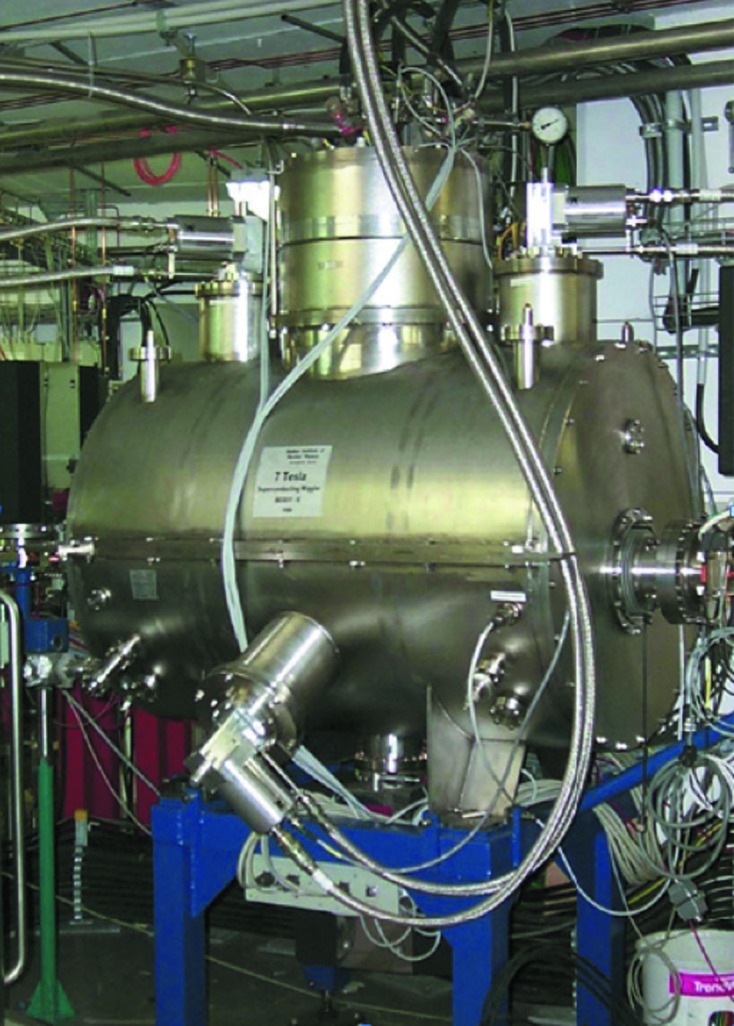
The 7T-WLS installed in the low-β section 14 of the BESSY II storage ring.

The source for all HZB MX beamlines BL14.1, BL14.2 and BL14.3 is also a superconducting 7T-WLS built at the Budker Institute. This 7T-WLS is installed within the low-β section 14 of the BESSY II ring (Table 1 ▶, Fig. 1 ▶). It serves as a very reliable and powerful X-ray point source and provides a very wide spectrum of X-rays from 0.1 keV to 100 keV with the maximum intensity at about 13.5 keV. Owing to the large 40 mrad horizontal radiation fan, all three HZB MX beamlines are fed from this single insertion device (ID).

### 2.3. The MX beamlines

**Figure 2:**
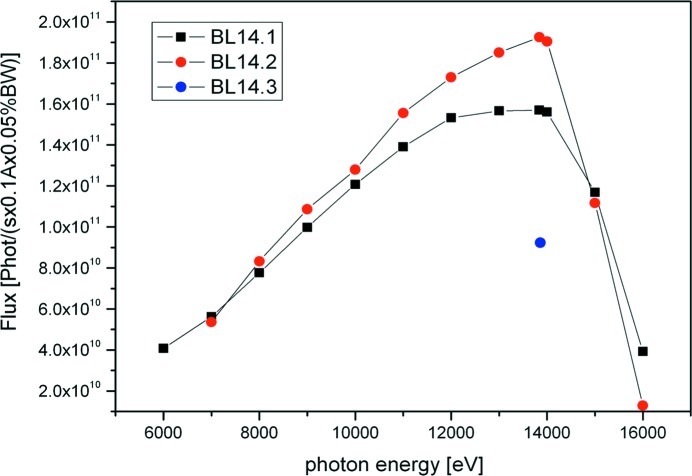
Photon flux of beamlines BL14.1, BL14.2 and BL14.3 as a function of energy.

Initially, the MX beamlines were funded, installed and set into operation by the first European structural genomics initiative ‘Protein Structure Factory’ (1999–2004) (Heinemann *et al.*, 2003 ▶). The beamlines were constructed and installed by ACCEL Instruments (now Bruker ASC, Bergisch-Gladbach, Germany). Over the past few years they have been developed to powerful work-horse beamlines for the *de novo* structure determination of currently more than 600 macromolecular structures deposited in the Protein Data Bank (PDB). At present, BL14.1 is operated and developed under the responsibility of HZB, while BL14.2 and BL14.3 are operated within a newly established consortium, the ‘Joint Berlin MX-Laboratory’, comprising the partners HZB, Freie Universität Berlin, Humboldt Universität zu Berlin, Max Delbrück Center Berlin and the Leibniz Institute for Molecular Pharmacology. The three MX beamlines have been differentially instrumented to support various experimental techniques. BL14.1 has recently been upgraded with an MD2 microdiffractometer including a mini-κ goniometer and an automated sample changer. It is therefore able to support the analysis of a large number of samples. It is also capable of supporting very small crystals down to 10 µm in the smallest dimension. BL14.2 provides a high-quality and high-intensity beam even at long wavelengths around 6 keV (Fig. 2 ▶). Owing to its very short minimum crystal–detector distance of 45 mm it is possible to collect large 2θ angles of up to 68° of diffracted X-rays at 0° 2θ offset. Consequently, BL14.2 can be used for various long-wavelength applications, including sulfur-SAD (S-SAD) (Mueller-Dieckmann *et al.*, 2005 ▶, 2007 ▶; Unge *et al.*, 2011 ▶) as well as atomic-resolution data collections (Gedrich *et al.*, 2010 ▶; Manjasetty *et al.*, 2005 ▶; Troyanov *et al.*, 2010 ▶). BL14.3 is a fixed-energy beamline, which is operated at 13.8 keV. At this energy it is possible to collect anomalous data from a large number of suitable heavy atoms, ranging from Zn (*K*-edge) to Pb (*L*_III_-edge). However, BL14.3 is mostly used as a general test beamline for the optimization of the diffraction properties of crystals with biophysical methods like controlled dehydration using the HC1c device (Sanchez-Weatherby *et al.*, 2009 ▶).

## 3. Beamline overview

### 3.1. Beamline optics

**Figure 3:**
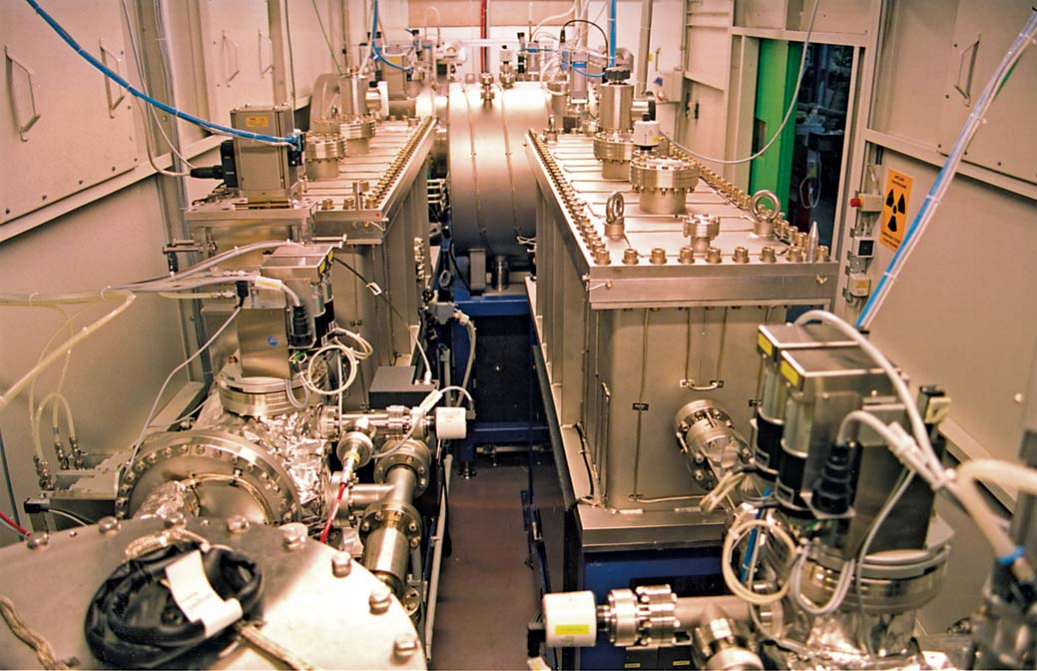
Upstream view of the joint optics hutch. BL14.2 and BL14.3 comprise the left part of the image, BL14.1 the right. The open green sliding door on the right-hand side is the only access to the optics hutch.

Since all three beamlines are fed from the same ID, they share the same optics hutch (Fig. 3 ▶). This is very economical with respect to the utilization of space, but requires a high level of organization.

**Figure 4:**
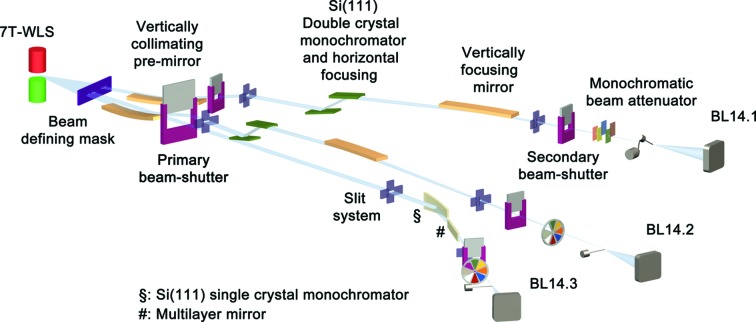
Schematics of the beamline layout for beamlines BL14.1, BL14.2 and BL14.3. The three beamlines are horizontally separated by the usage of the 40 mrad broad synchrotron beam fan. The BESSY storage ring wall is located at the downstream side of the primary beam-shutter.

The tunable-energy beamlines BL14.1 and BL14.2 consist of two front-end sections including a vertically collimating Si mirror coated with a 50 nm Rh layer and a primary beam-shutter. In the optics hutch an indirectly water-cooled Si(111) double-crystal monochromator with horizontal focusing *via* sagittal bending of the second monochromator crystal and a vertically focusing mirror coated with a 50 nm Rh layer are installed. A secondary beam-shutter is located at the end of the beamline (Fig. 4 ▶). Several beam-position, beam-shape and beam-intensity monitors are installed within the beam path of both beamlines, which provide all necessary information to analyze and evaluate the beam quality and the beam position at any time (Fuchs *et al.*, 2007 ▶).

BL14.3 shares within the front-end section the primary beam-shutter with BL14.2. Inside the optics hutch it consists of an asymmetrically cut Si(111) single-crystal monochromator with direct water cooling and a meridional bender for horizontal focusing, adjusted to an energy of 13.8 keV. A horizontally deflecting lateral gradient and cylindrically shaped multi-layer mirror made from 200 Si/Mo bilayers for vertical focusing of the monochromatic X-ray beam and a secondary beam-shutter complete the set-up.

### 3.2. Beamline end-stations

#### 3.2.1. BL14.1

**Figure 5:**
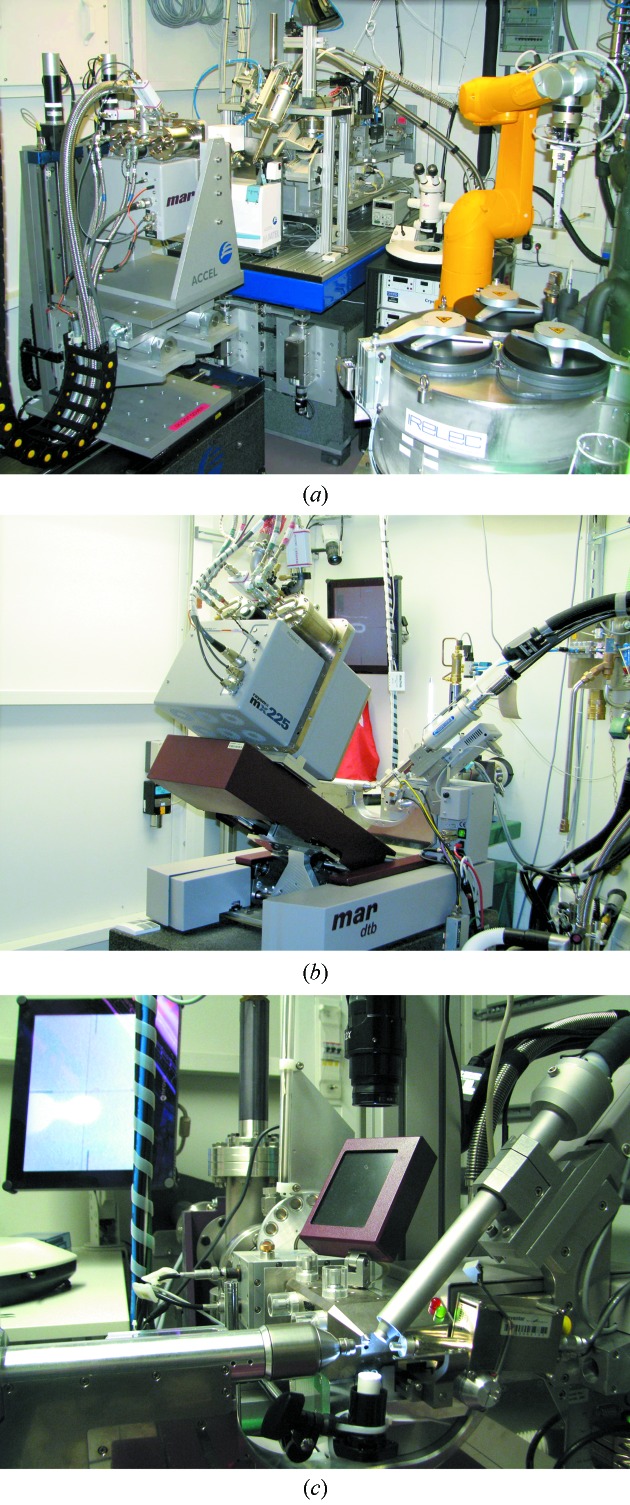
Experimental stations instrumentations: (*a*) BL14.1, (*b*) BL14.2, (*c*) BL14.3.

Beamline BL14.1 was significantly upgraded recently and is currently the most automated and advanced of the three HZB MX beamlines (Table 2 ▶, Fig. 5*a* ▶). The end-station is equipped with a microdiffractometer MD2 with ready-to-use mini-κ MK3 (Fig. 7*e*) (Maatel, Voreppe, France). Integrated with the MD2 is a CATS (Cryogenic, Automated Transfer System, Irelec, Grenoble, France) sample changer, which allows the user to analyze up to 90 cryo-cooled SPINE-compatible (Cipriani *et al.*, 2006 ▶) samples or crystals from four SBS-compatible 96-well crystallization plates in the experimental hutch without entering the hutch. The detector installed in the endstation of BL14.1 is a Rayonix 225 mm (Rayonix, Evanston, Illinois, USA) 3 × 3 tiled CCD device. This detector can be moved within a distance range between 93 mm and 730 mm from the sample. A 2Θ stage supports a detector-angle offset of up to 20°, to accept large reflection orders at long detector distances. Further equipment comprises an X-Flash fluorescence detector (Bruker AXS, Berlin, Germany) for elemental X-ray absorption edge scans and X-ray-fluorescence-based element identification. A remotely controlled cryo-shutter is available on BL14.1 as well as on the other two beamlines, which allows the automated cryo-annealing of cryo-cooled samples. A permanently installed pulsed UV laser (λ = 266 nm) produces highly intense radiation, which can be coupled to a crystal *via* a 3 m-long quartz fiber for UV-RIP experiments (Schönfeld *et al.*, 2008 ▶). At all three beamlines the crystal cooling to temperatures between 95 K and 280 K is achieved with a Cryojet-XL (Agilent Technologies, Oxford, UK). All liquid-nitrogen-requiring devices are directly connected to the central liquid-nitrogen storage vessel of BESSY II and are refilled in a fully automatic mode.

#### 3.2.2. BL14.2

The experimental station of BL14.2 consists of a mardtb diffractometer (Marresearch, Norderstedt, Germany) with a 2Θ stage, which supports a 2Θ angle offset of up to 30°. A Rayonix 225 mm CCD detector and an X-Flash fluorescence detector are installed here as well (Table 2 ▶, Fig. 5*b* ▶). The CCD-detector-to-crystal distance can be varied between 45 mm and 380 mm. The possibility to use very short crystal-to-detector distances is of particular importance for the high-resolution diffraction experiments at low X-ray energies. The same is true for the determination of atomic- and subatomic-resolution structures of macromolecules and small molecules. Without using the 2Θ angle offset the highest resolvable resolution by the circular area of the CCD is 0.7 Å at 13.5 keV X-ray energy and 1.9 Å at 5.9 keV X-ray energy.

#### 3.2.3. BL14.3

The experimental station of BL14.3 consists of a mardtb diffractometer with the same functionality and geometries as BL14.2. The detector installed on BL14.3 is a Rayonix SX165 CCD detector (Table 2 ▶, Fig. 5*c* ▶). Alternative to the cooling nozzle, a HC1c (Maatel, Vorreppe, France) crystal dehydration device can be installed. This device is used to alter the crystal hydration levels in a controlled and reproducible manner in order to improve the diffraction properties of the crystal under investigation (Sanchez-Weatherby *et al.*, 2009 ▶; Russi *et al.*, 2011 ▶).

### 3.3. Additional beamline infrastructure

#### 3.3.1. Beamlines and end-station control systems

The motors of the beamline components of all MX beamlines are operated by VME-based motor controllers of type OMS-58 (Oregon Micro Systems, Beaverton, USA). Each of the three beamlines is controlled independently by the *SPEC* instrument-control software (Certified Scientific Software, Cambridge, USA), which also provides a software interface for remotely controlled operation by upper-level graphical user interfaces (GUIs). The end-station of BL14.1 uses a Delta Tau PMAC motor controller for the experimental table and the MD2 as well as a CS8C controller for the CATS sample changer. All end-station components provide software interfaces for remote control, either using the TACO/TANGO protocol (experimental table, MD2, Rayonix CCD detector) or a proprietary network-socket API (CATS). The quite diverse control systems are combined and managed by the Bliss Framework hardware abstraction layer (Guijarro *et al.*, 2004 ▶), which provides a central software interface to the devices irrespective of their native control systems. At the user level, the *MxCuBE* beamline GUI (Gabadinho *et al.*, 2010 ▶) enables the users to perform beamline-specific tasks as well as the set-up of the diffraction experiment and X-ray fluorescence, analysis and absorption edge scans. The Bliss Framework and the *MxCuBE* GUI have been installed and customized in close collaboration with the ESRF Bliss group (now BCU/Software Group) and will be further developed within the framework of the recently founded *MxCuBE* collaboration between several European synchrotron facilities. On BL14.2 and BL14.3 two separate control programs are used during normal operation: beamline-specific tasks (monochromator control, beam attenuation, acquisition of fluorescence data for optimized MAD data-collection strategies) are controlled by an in-house-developed GUI whereas the set-up and operation of the diffraction data experiment is controlled by the vendor-supplied CCD-detector control program *marccd* (Rayonix, Evanston, Illinois, USA). The HC1c installed on BL14.3 is operated by the control software provided by Maatel.

#### 3.3.2. Data processing infrastructure

Each beamline is equipped with a dedicated 12-core high-performance data-evaluation server. Each of these servers is connected *via* two 4 Gbit interfaces to a HP EVA-SAN system to data storage containers, which are flexible in size. In order to guarantee data safety and integrity, all data are automatically backed-up on a daily basis and are stored for 6 to 12 months on SDLT tapes, which is organized by the *EMC-Networker* software. A new 40-core server machine has recently been installed to extend the number-crunching performance of our data-evaluation platform. Users are able to process their diffraction data on-site using *XDS* (Kabsch, 2010 ▶) and *iMosflm* (Battye *et al.*, 2011 ▶).

For *XDS* we are developing two upper-level expert control scripts named ixds and xdsapp, which facilitate the set-up and the decision-making process during *XDS* data processing (Krug *et al.*, 2012 ▶). With this hardware and software it is easy to follow all data collection on-the-fly. This means that it is possible to obtain feedback during the experiment about the quality of the diffraction data as well as 1 to 2 min after the completion of the diffraction experiment for all collected data.

#### 3.3.3. Experiment control room

**Figure 6:**
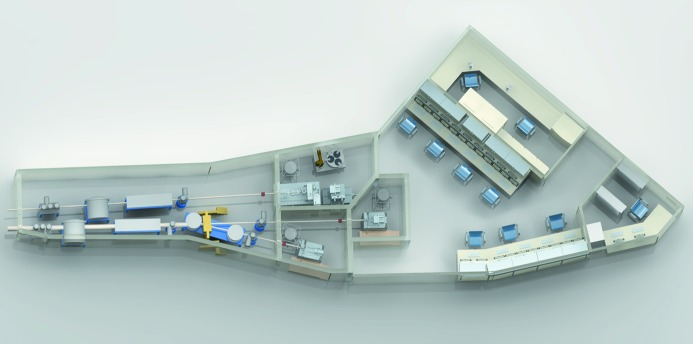
Three-dimensional drawing of the MX beamline optics (left) and experimental hutches (centre) as well as the experiment control room and sample preparation laboratory (upper right). Beamline order from top to bottom: BL14.1, BL14.2, BL14.3.

The experiment control room, from which all experimental stations are controlled by the experimenters, is directly attached to the experimental hutch entrances. This room creates a noise-reduced working environment for the researchers and offers all required resources from experimental control stations to data processing and back-up terminals up to the sample preparation laboratory. Access to this room is limited to users for the time of their experiment and to beamline staff (Fig. 6 ▶).

#### 3.3.4. Automated monitoring system

All beamline components as well as many experimental station components including the control PCs are incorporated into an automatic monitoring system, which informs the responsible staff in case of any deterioration of the beamline performance *via* cell phone voice and text messages. The uninterrupted and steady surveillance of these components over the past eight years is one of the keys to the extremely low unscheduled system downtimes as well as the long-term steady delivery of a high-quality X-ray beam to our experimental facilities.

## 4. Ancillary facilities

**Figure 7:**
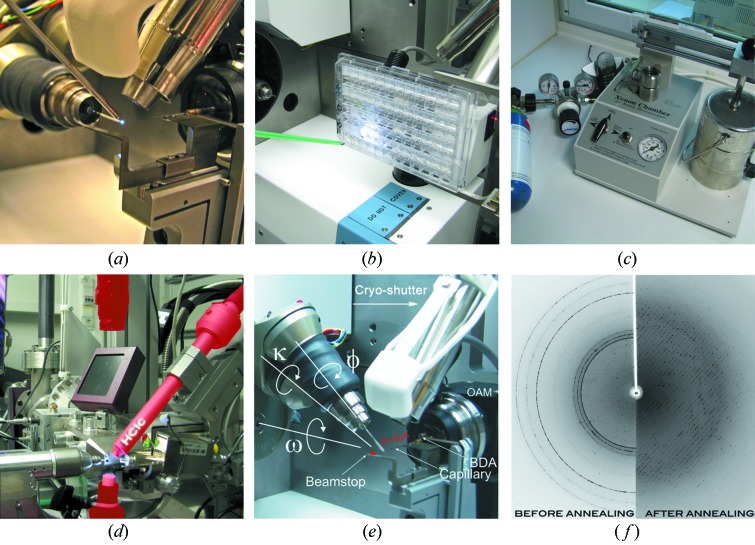
Ancillary facilities of the MX beamlines. (*a*) UV-RIP set-up on BL14.1. The crystal which is exposed to UV irradiation can easily be seen owing to its fluorescence, (*b*) *In situ* crystal screening of 96-well plates centred within the X-ray beam of BL14.1. (*c*) Noble gas pressure cell for Xe and Kr derivatizations (http://www.hamptonresearch.com/). (*d*) HC1c dehydration device mounted on BL14.3. (*e*) Cryo-shutter annealing device at BL14, with microdiffractometer MD2 and mini-κ MK3. (*f*) Results from the cryo-shutter operation (the device mounted on all beamlines); the image shows a diffraction pattern before (left) and after (right) cryo-annealing.

In addition to the set-up at the three HZB MX beamlines, ancillary facilities are available, which allow users and in-house staff to perform specialized experiments. These facilities will be described in the following section (Fig. 7 ▶).

### 4.1. UV-RIP

The UV-RIP (ultraviolet radiation-damage-induced phasing) method has been developed recently at EMBL-Grenoble by Raimond Ravelli and co-workers (Nanao & Ravelli, 2006 ▶). It is based on specific structural changes in cystine-containing protein crystals, which are induced by irradiation with highly intensive UV radiation. The structural changes can be used to work out a ‘single isomorphous replacement’-like phasing scheme (Nanao *et al.*, 2005 ▶), which can lead to precise experimental phase information. In order to perform the experiment, a low-dose native dataset is collected and compared with another low-dose dataset collected after the UV irradiation of the same crystal (Faust *et al.*, 2010 ▶). This method may have considerable potential as an alternative way of experimental phasing, but, owing to the novelty of this method, the success rate for UV-RIP phasing for real-case proteins still has to be determined (Schönfeld *et al.*, 2008 ▶). For this reason we are offering interested academic users the appliance of our UV-RIP instrumentation at BL14.1 (Fig. 7*a* ▶) and the assistance from our beamline staff to provide support during the experiment as well as for the data evaluation.

### 4.2. *In situ* crystal screening

The experimental end-station of BL14.1 can be used to perform *in situ* screening of crystalline samples (Fig. 7*b* ▶). With this technique it is possible to expose a crystal inside its native crystallization drop to X-rays utilizing the CATS robotic arm as a goniometer device (Jacquamet *et al.*, 2004 ▶). The diffraction images acquired can then be indexed to obtain information about various crystal properties such as unit-cell parameters, crystal symmetry and crystal mosaicity. This is valuable information for selecting optimal crystallization conditions. The method can be further utilized to differentiate between macromolecular and salt crystals (Paithankar *et al.*, 2012 ▶).

### 4.3. Noble gas derivatization

The Hampton Research (Aliso Viejo, USA) noble gas chamber is located in the sample preparation laboratory and can be used to produce xenon and krypton noble-gas derivatives of macromolecular crystals (Fig. 7*c* ▶) to assist in phase determination or to map solvent channels within protein crystals (Gabdulkhakov *et al.*, 2009 ▶; Moschetti *et al.*, 2009 ▶).

### 4.4. HC1c crystal dehydrator

Many biological macromolecules yield crystals which display significant inherent disorder, and as a consequence diffract X-rays rather poorly. One approach to increase the crystalline order is to dehydrate the crystal in a controlled manner before shock-cooling it to 100 K for storage or for data collection. One of the first commercial devices for this was the free-mounting system (FMS; Rigaku Americas, The Woodlands, USA) developed by Kiefersauer and colleagues (Kiefersauer *et al.*, 2000 ▶). However, owing to its rather difficult and cumbersome operation, the FMS never became very popular in the MX community. Recently, a new device (HC1) has been developed by Sanchez-Weatherby and colleagues at the EMBL-Grenoble, which allows for a much easier use (Sanchez-Weatherby *et al.*, 2009 ▶; Russi *et al.*, 2011 ▶). Since January 2011, an HC1c device has been installed on BL14.3 (Fig. 7*d* ▶). It is very easy to operate and enables the user to improve the diffraction limit of a crystal by controlled dehydration, to determine optimal cryo-cooling conditions, and to determine optimal soaking conditions for macromolecular crystals. The device can be used to perform data collections at ambient temperature and without surrounding mother liquor as well. For dehydration experiments it is necessary to bring crystals in crystallization trays. Furthermore, the crystals need to be mountable on micro-meshes. Since HC1c experiments require some changes to the beamline set-up, it is essential to apply for its usage in advance.

### 4.5. Crystal annealing

Cryogenic data collection is a tight-rope walk between lowering radiation damage and increasing mosaicity owing to lattice dis­ordering produced during cooling of the crystal. Cryo-annealing can significantly reduce the mosaicity of the crystals and can also improve diffraction of flash-cooled crystals (Fig. 7*f* ▶) that were mistreated during transfer to and from cryogenic storage (Harp *et al.*, 1999 ▶). The set-up of the HZB MX beamlines allows a reliable and time saving application of this method by providing a remotely controlled cryo-annealing shutter on all three beamlines (Fig. 7*e* ▶). With this in-house-designed device, which is based on a three-dimensional printed flexor design polyamide structure (Mueller *et al.*, 2012 ▶), it is possible to block the cryostream for a pre-defined time. In order to further investigate the principle of cryo-annealing, we started a long-term study to unravel the role of the different factors that influence this method. Owing to the unique construction and miniaturization of the device, several international MX facilities are interested in utilizing it or are already using this device, such as the Australian Synchrotron, Diamond Light Source and the Swiss Light Source.

## 5. Facility access and user community

Access to the HZB MX beamlines is provided *via* the online access tool *GATE* (http://www.bessy.de/boat/www/). *GATE* is the electronic platform for the complete user communication in order to register, apply for beam time, manage given beam time resources and the user-controlled scheduling of all MX beamlines utilizing the *GATE* PX calendar. This electronic calendar opens the possibility to analyze the booking situation for all beamlines and to book individual experimental beam times during the lifetime of the proposal. Twice per year, new proposal rounds are organized by HZB. Following the submission of individual user proposals, an external beam time committee for MX, consisting of four highly regarded international experts, evaluates the quality and the feasibility of all proposals and ranks them in order to maximize the user output at the beamlines.

## 6. Teaching activities

Despite the growing importance of MX in basic biological and medical research, methods in MX are inadequately reflected in most university curricula around the world (Faust *et al.*, 2008 ▶; Jaskólski, 2001 ▶). This has led to the fact that the majority of synchrotron users are life science experts, biochemists or molecular cell biologists, with little or no training in condensed matter physics including X-ray diffraction methods. Consequently, the question needs to be answered, how in particular new members of the MX community can be put in a situation to be able to perform successful diffraction data collection and structure solution using synchrotron radiation? At the HZB, the MX group is investing considerable resources to educate the community on a regular basis using complementary approaches. Our group is one partner of a multi-module master students course of the Department of Biology, Chemistry and Pharmacy of the Freie Universität Berlin. Within this module the students learn how to collect X-ray diffraction data for *de novo* structure solutions, like MAD, S-SAD, SIRAS and UV-RIP, of several proteins of choice with hands-on-experiences using all of our MX beamlines. A second tier is a course for PhD students on ‘Diffraction data collection using synchrotron radiation’, which is organized every second year as an educational initiative of the biocrystallography working group of the German Crystallographic Society, DGK. During this workshop 20 novice students from all over the world, who are masters and PhD students, have their first practical experiences using our MX beamlines under the guidance of eight international tutors. As a spin-off from these workshops we have developed over the past years the ‘X-ray tutorial for learning and teaching macromolecular crystallography’ (Faust *et al.*, 2008 ▶, 2010 ▶). This unique collection of experimental procedures from crystallization to data collection and structure determination comprises seven different representative case studies for modern X-ray crystallography. The tutorial is online and freely available from our webpage (http://www.helmholtz-berlin.de/bessy-mx).

## 7. User experiments

### 7.1. Deposition statistics and user output

**Figure 8:**
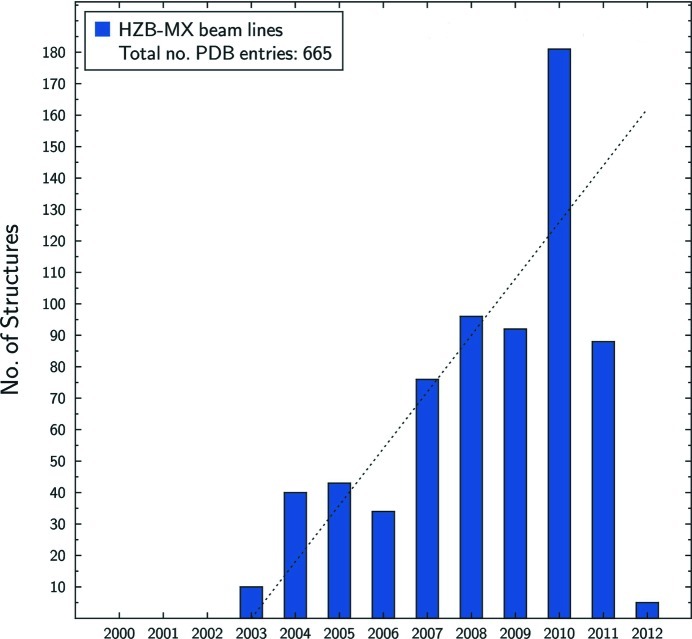
Histogram of released protein structure coordinates per year from the PDB (data were taken from the BioSync web page, http://biosync.sbkb.org/). As a consequence of the 12-months hold on deposited structures, the deposition numbers for 2011 will only be finalized by the end of 2012. Status from 9 February 2012.

Currently, about 50 research groups from non-profit research organizations and industry are actively and continuously using the HZB MX facilities. Twice a year, all users are requested to submit their scientific application to the review panel. Currently, the submitted MX proposals comprise approximately 20% of all photon science proposals submitted to HZB. Resulting from the evaluated scientific cases and the successful experiments at our MX-beamlines, more than 600 structure depositions to the PDB have been made over the past years of operation (Fig. 8 ▶). A recently conducted survey provided further information about the actual beamlines output of our facilities. Within the past three years of operation, besides the 361 PDB depositions, the scientific output comprises 358 scientific papers, including 19 in high-impact-factor journals, 103 PhD theses and 57 master and diploma theses.

### 7.2. Research highlights

#### 7.2.1. Opsin, the activated form of the GPCR rhodopsin

**Figure 9:**
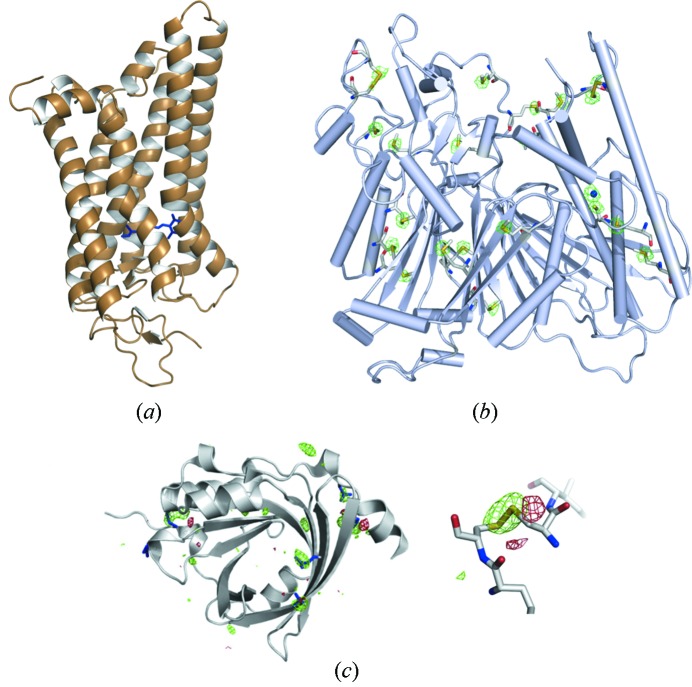
Representative examples of research highlights from diffraction data collected at the HZB MX beamlines. (*a*) Meta II rhodopsin structure (Choe *et al.*, 2011 ▶), (*b*) 66.3 kDa protein structure solved by S-SAD (Lakomek *et al.*, 2009 ▶), (*c*) α1-acid glycoprotein solved by UV-RIP with highlighted disulfide bonds and contoured +4.5σ and −4.5σ (green and red, respectively) *F*_before_ − *F*_after_ difference density (Schönfeld *et al.*, 2008 ▶).

Rhodopsin is a so-called G-protein coupled receptor or GPCR. GPCRs are seven-helix membrane proteins which are very important members of signal transduction pathways and primary targets for the pharmaceutical industry. Scientists from the biophysical institute of the Charité, University Medicine Berlin, managed to gain structural insights into the ligand-free form of this GPCR, the opsin (Park *et al.*, 2008 ▶). It was also possible to determine the structure of opsin in complex with a signaling peptide of the corresponding G-protein (Scheerer *et al.*, 2008 ▶) and of the light activated form, the all-trans-retinal bound metarhodopsin II (Choe *et al.*, 2011 ▶) using BL14.2 (Fig. 9*a* ▶).

#### 7.2.2. Lysosomal 66.3 kDa protein solved by S-SAD

This 66.3 kDa large hydrolase from mouse consists of 559 amino acids and has no considerable sequence similarity with other proteins of known three-dimensional structure. The polypeptide chain has 22 bound S atoms, which made it possible to solve this protein structure by S-SAD phasing at BL14.2 of BESSY to a maximum resolution of 2.4 Å using 1.9 Å X-ray irradiation wavelength (Fig. 9*b* ▶). This 66.3 kDa protein is one of the largest S-SAD structures which has been solved so far and demonstrates the potential of this long-wavelength phasing method (Lakomek *et al.*, 2009 ▶).

#### 7.2.3. α1-Acid glycoprotein (AGP) solved by UV-RIP

The structure of the unglycosylated human AGP has been determined to a final resolution of 1.8 Å by the UV-RIP phasing method (Fig. 9*c* ▶). The 183-amino-acid-containing protein has two disulfide bridges which were photolysed to produce phase information on the basis of the conformational change of the free cysteine side chains (Schönfeld *et al.*, 2008 ▶). To our knowledge this structure determination was one of the first successful applications of the UV-RIP method to a hitherto unknown protein structure.

## 8. Discussion and conclusions

At the Berlin electron storage ring BESSY II of the HZB in Berlin, three MX beamlines are operated successfully. Two of them are energy tunable over a large energy range, one is a fixed-energy beamline. Besides the MAD capability of BL14.1 and BL14.2, the HZB MX beamlines can be used to screen large sample ensembles within a short period of time, to determine the diffraction properties of crystals within crystallization plates and for the *de novo* structure solution using long-wavelength applications like S-SAD. BL14.1, with its microdiffractometer and mini-κ, offers multi-axis goniometry and the data collection from small crystals down to 10 µm sample length. BL14.2 can be used to collect long-wavelength or atomic-resolution data and BL14.3 is used as a screening beamline to improve the diffraction properties of protein crystals. Ancillary equipment, *e.g.* the dehydration control device HC1c, a noble gas chamber, the UV-RIP set-up and the crystal annealing devices, can be used to carry out successful structure solution experiments.

Over the past years of operation more than 665 new protein structures have been deposited to the PDB by our user community. Within the past three years a steady increase in output resulted in 356 user publications, 361 structure depositions and 103 completed PhD theses using our beamlines.

Various teaching activities are organized by the MX beamline scientists to continuously improve the quality of X-ray data collection by the user community.

## Figures and Tables

**Table 1:** Characteristics of the electron storage ring BESSY II and the 7T-WLS source

Electron energy	1.7 GeV
Circumference	240 m
Emittance	5 nrad m
Magnetic field	7 T
Critical energy	13.8 keV
Used horizontal opening	40 mrad
Source size (σ_*x*_)	50 µm
Source size (σ_*y*_)	20 µm
Source divergence (σ_*y*′_)	21 µrad

**Table 2:** Primary characteristics of the three HZB MX beamlines

	BL14.1	BL14.2	BL14.3
Wavelength range (Å)	0.8–2.25	0.8–2.25	0.89
Maximum intensity (Å)	0.92	0.92	–
Photon flux at sample (uncollimated) [photons s^−1^ (0.1 A)^−1^ (0.05% bandwidth)^−1^]	1.3 × 10^11^	1.9 × 10^11^	4 × 10^10^
Energy resolution (eV)	< 2	< 2	< 5
Sample automation	CATS sample mounting robot (handling of up to 90 SPINE-standard samples and 96-well plates)	–	–
Goniometry	MD2 microdiffractometer with MK3 mini-κ	mardtb	mardtb
X-ray detector	Rayonix MX-225	Rayonix MX-225	Rayonix SX-165
Beam size (collimated) (µm)	30–100 (diameter)	150 × 100 (h × v)	200 × 100 (h × v)
Achievable resolution (Å)	0.9	0.7	0.9
Maximum unit-cell length (at 2.0 Å maximum resolution) (Å)	400	400	250
Typical exposure time (s)	0.2–10	3–10	3–30
Special equipment and operations	*In situ* crystal screening; UV-RIP; three-axis gonio­meter; on-axis zoom microscope; crystal annealing; HT-crystal screening	Very short detector–sample distance (45 mm); crystal annealing; ultra-high-resolution data collection; long-wavelength data collection	Crystal diffraction improvement by controlled dehydration with HC1c; crystal annealing; crystal screening
